# Supplementary material for: Identification of a non-axisymmetric mode in laboratory experiments searching for standard magnetorotational instability
Source: Nat Commun. 2022 Aug 9;13:4679. doi: 10.1038/s41467-022-32278-0 (PMC9363437; doi:10.1038/s41467-022-32278-0)
Supplement: Supplementary file 1 — Supplementary Information [file 41467_2022_32278_MOESM1_ESM.pdf]

Supplementary Information for
Identification of a non-axisymmetric mode in laboratory experiments searching for standard magnetorotational instability

Yin Wang[1*], Erik P. Gilson[1], Fatima Ebrahimi[1,2], Jeremy Goodman[2], Kyle J. Caspary[1], Himawan W. Winarto[2], and Hantao Ji[1,2]
[1]*Princeton Plasma Physics Laboratory, Princeton University, Princeton, New Jersey 08543, USA*
[2]*Department of Astrophysical Sciences, Princeton University, Princeton, New Jersey 08544, USA*

This PDF file includes:

Supplementary Fig. 1. An example of radial magnetic field variations as a function of azimuth angle θ.
Supplementary Fig. 2. Spectral analysis of the hydrodynamic Rayleigh instability.
Supplementary Fig. 3. Azimuthal structure of hydrodynamic Rayleigh instability.
Supplementary Fig. 4. Space domain of the simulation.
Supplementary Fig. 5. Time evolution of the flow field.
Supplementary Fig. 6. Spatial distribution of azimuthal magnetic field.

* ywang3@pppl.gov

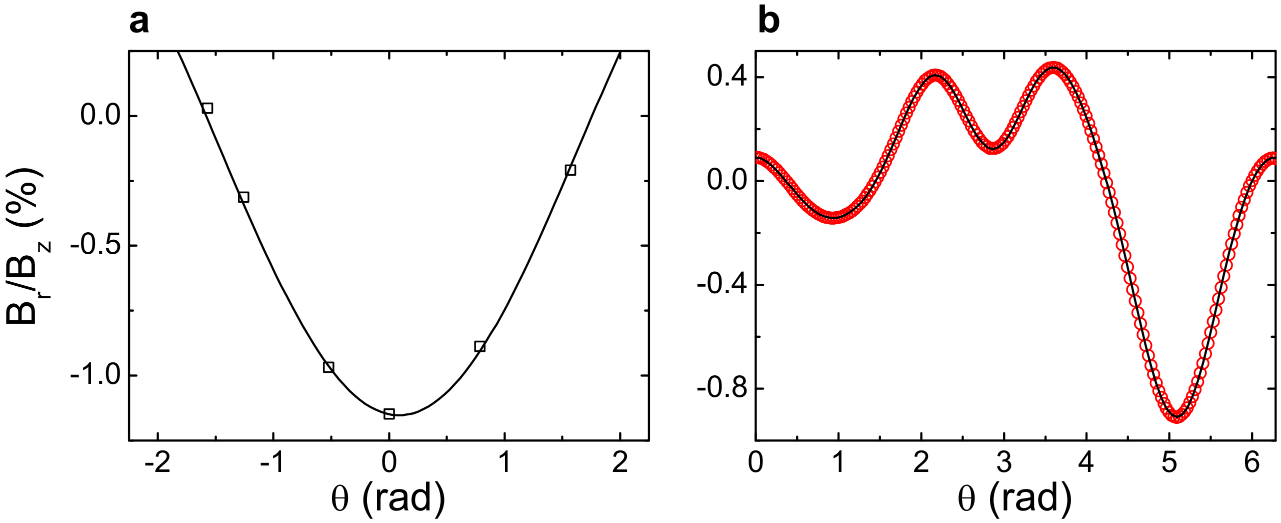

Supplementary Fig. 1. **An example of radial magnetic field variations as a function of azimuth angle θ.** The measurement is made in the midplane and at the inner cylinder surface for experiment (**a**) and simulation (**b**) at Rm $= 3$ and $B_0 = 0.2$. Solid lines are fits of Eq. (2) in the main text to the data points with $N = 2$ (**a**) and $N = 10$ (**b**).

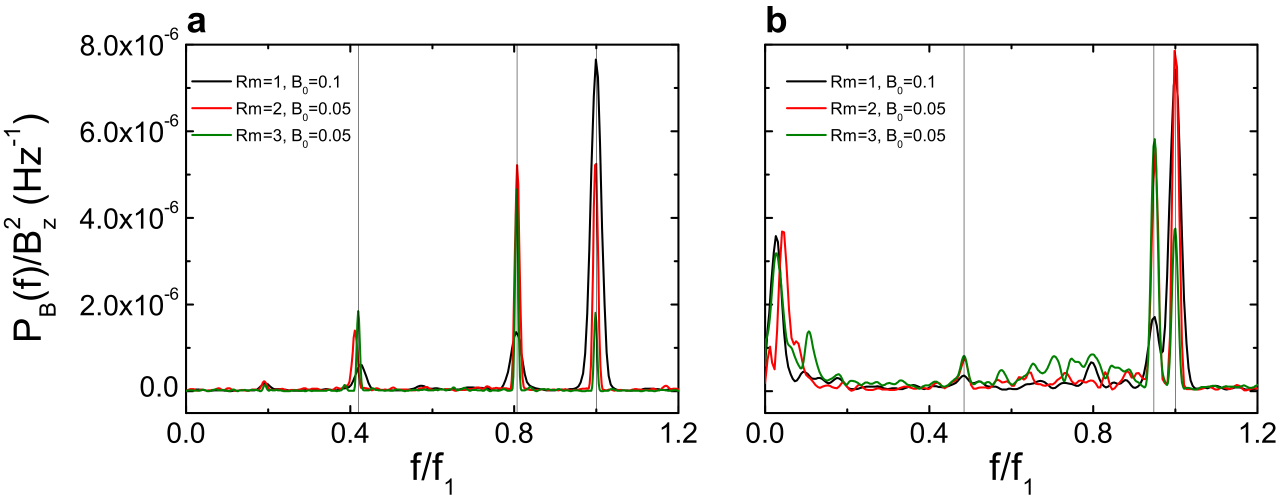

Supplementary Fig. 2. **Spectral analysis of the hydrodynamic Rayleigh instability**. Measured normalized power spectrum $P_B(f)/B_z^2$ as a function of normalized frequency f/f_1. The measurements were made in the midplane with various Rm in the presence of a weak magnetic field. The angular velocity ratio was fixed at $\Omega_1 : \Omega_3 : \Omega_2 = 1 : 0.58 : 0.19$ (**a**) and $\Omega_1 : \Omega_3 : \Omega_2 = 1 : 0.507 : 0.05$ (**b**). In both panels, the vertical lines from left to right represent the machine-induced dimensionless frequency $1 - f_3/f_1$, $1 - f_2/f_1$ and 1.

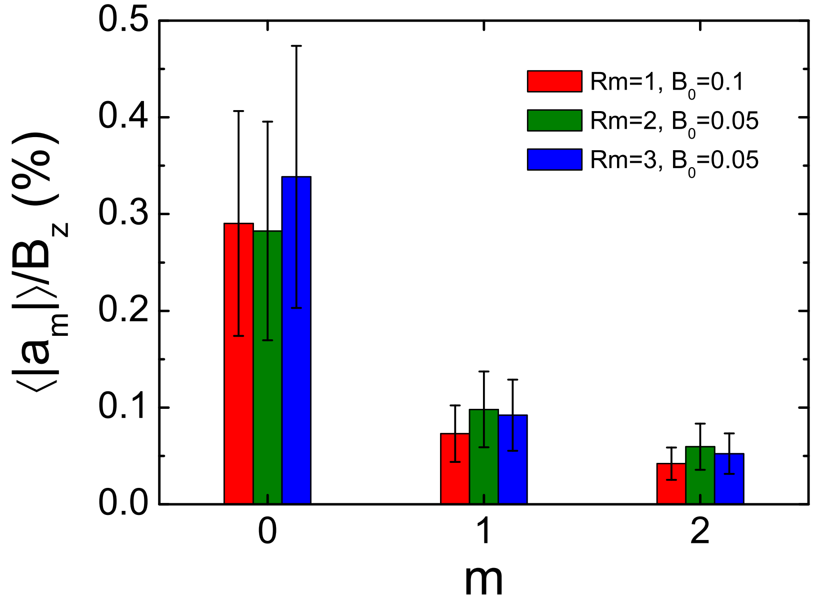

Supplementary Fig. 3. **Azimuthal structure of hydrodynamic Rayleigh instability.** Measured normalized mode amplitudes $\langle|a_m|\rangle/B_z$ in the experiment with the Rayleigh unstable configuration, as a function of azimuthal mode number m for different values of Rm with a weak magnetic field. The measurements are made in the midplane and at the inner cylinder surface. The error bars show the standard deviation.

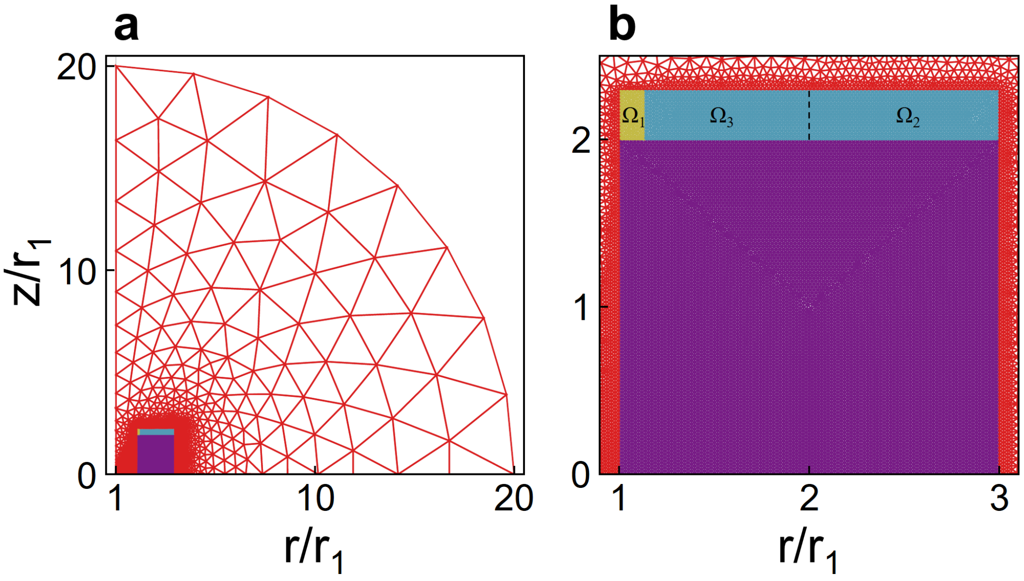

Supplementary Fig. 4. **Space domain of the simulation.** Mesh arrangement in a quarter section of the meridional plane (**a**) and an enlarged portion around the Taylor-Couette cell with rotational speeds of different components marked (**b**) . Colors indicate different domains with purple as the fluid, blue as the copper endcap rings, yellow as the stainless steel rim of the inner cylinder, and red as the vacuum. The black dashed line in (**b**) indicates the boundary between the inner and outer rings. This plot was created by the authors and previously published [Winarto, H. et al., *Phys. Rev. E* **102**, 023113 (2020)].

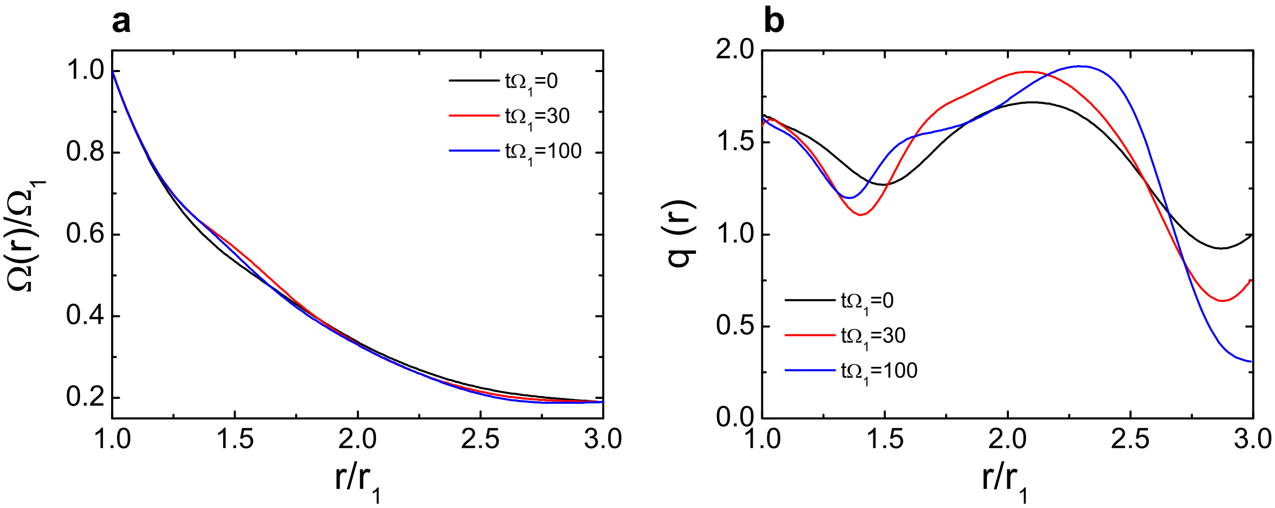

Supplementary Fig. 5. **Time evolution of the flow field.** Numerically calculated angular velocity profile $\Omega(r)$ (**a**) and corresponding $q \equiv -\partial \ln\Omega(r)/\partial \ln r$ profile (**b**) at different times (t) after the imposition of the axial magnetic field. The calculation is made at $\mathrm{Rm} = 6$ and $B_0 = 0.2$, and averaged azimuthally and vertically in the bulk region with $-0.25 \leq z/H \leq 0.25$.

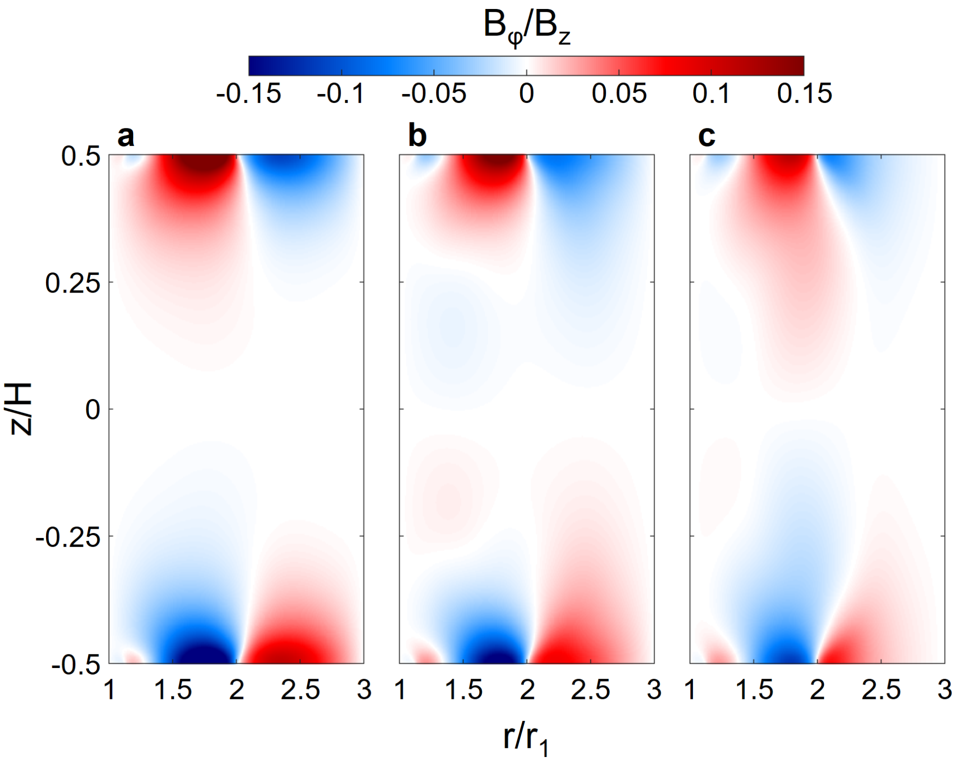

Supplementary Fig. 6. **Spatial distribution of azimuthal magnetic field.** Normalized azimuthal magnetic field B_ϕ/B_z in the meridional plane, which is obtained from 3D simulation at fixed Rm = 4 and different values of B_0: (**a**) $B_0 = 0.05$, (**b**) $B_0 = 0.2$ and (**c**) $B_0 = 0.4$. The calculation is based on time and azimuthal averages in the MHD steady state.
