# Supplementary material for: Identification of a non-axisymmetric mode in laboratory experiments searching for standard magnetorotational instability
Source: Nat Commun. 2022 Aug 9;13:4679. doi: 10.1038/s41467-022-32278-0 (PMC9363437; doi:10.1038/s41467-022-32278-0)
Supplement: Supplementary file 3 — Description of Additional Supplementary Files [file 41467_2022_32278_MOESM3_ESM.pdf]

File name: Supplementary Movie 1

Description: Time evolution of the radial magnetic field in the midplane from simulation at $Rm = 6$ and $B_0 = 0.2$.
